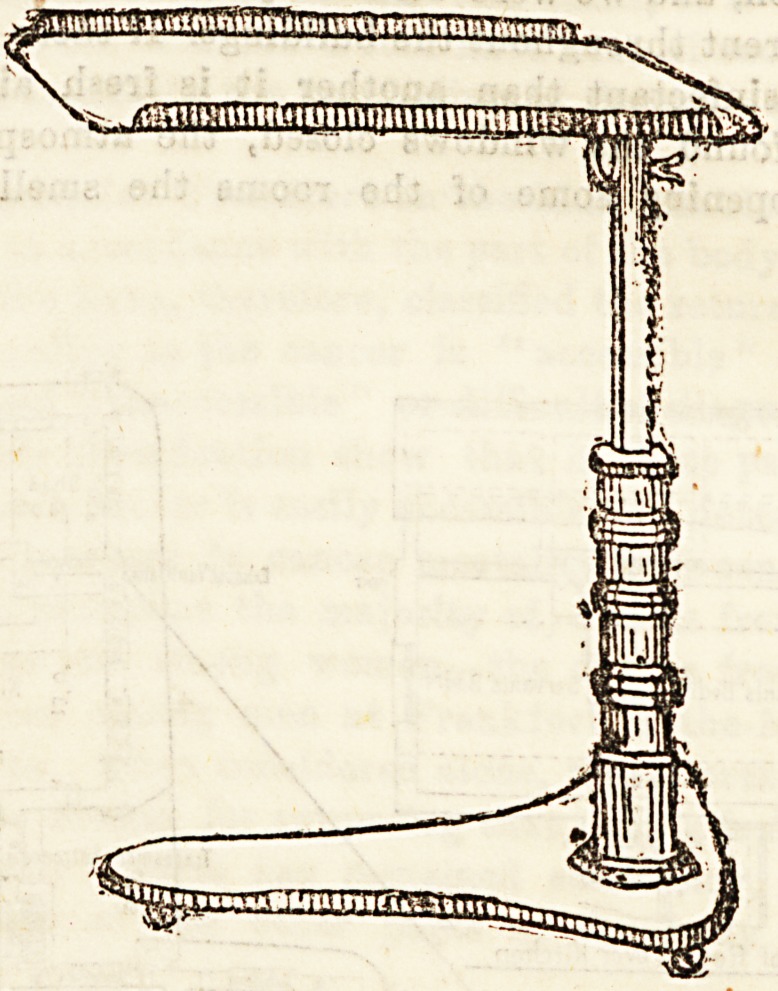# Bed Tables

**Published:** 1893-05-20

**Authors:** 


### PRACTICAL DEPARTMENTS.

### BED TABLES.

A great addition to invalid comfort is a really convenient bed-table. When made to combine the purposes of a tray for meals and a book-rest, as shown in our first illustration, they are an improvement upon those generally used in hospitals, and these can be procured from Messrs. Farmer, Lane, and Co., 77, 79, New Oxford Street, at a very small extra cost. The desk part, which is adjustable to any height, will close down to enable the table to be used for meals, &c. The legs are made to unscrew, a great convenience when it is a question of travelling or economy of space, as when these are removed the table will pack into quite a small parcel. We would make one suggestion, and that is that if the legs were somewhat heavier in make it would add to the practical value of the table, even if its appearance became thereby less attractive. Steadiness is a very essential point, and it will easily be seen that when the legs end in a point it is not so easy for the balance to be kept, and the table is thus apt to tilt to one side. These little tables are made in pine, birch, mahogany or oak. Very useful little reading desks may be had separately, if preferred, to place on the table. These also have legs to screw in, so thus the height may be altered as desired. Messrs. Farmer, Lane, and Co. have also what is described as a "meal-carrier"; that is to say, a tray with handles, which is provided with moveable legs, and can thus be utilized as tray and bed-table combined.

Our second illustration shows a "pillar" bed-table. As will be seen, these are made to project over the bed, and are cleverly contrived to suit every position. They are quickly and easily altered to any height, and the tray can be tilted and used as a reading desk. Some of them are made with rising desks. The prices are really very moderate. Delightful reading stands, some provided with shaded lamp or candle, others to hold two or three books at once, and so arranged as to be conveniently adjusted for reading in a recumbent position, may be seen at Messrs. Farmer and Lane's show-rooms.

## Figures and Tables

**Figure f1:**
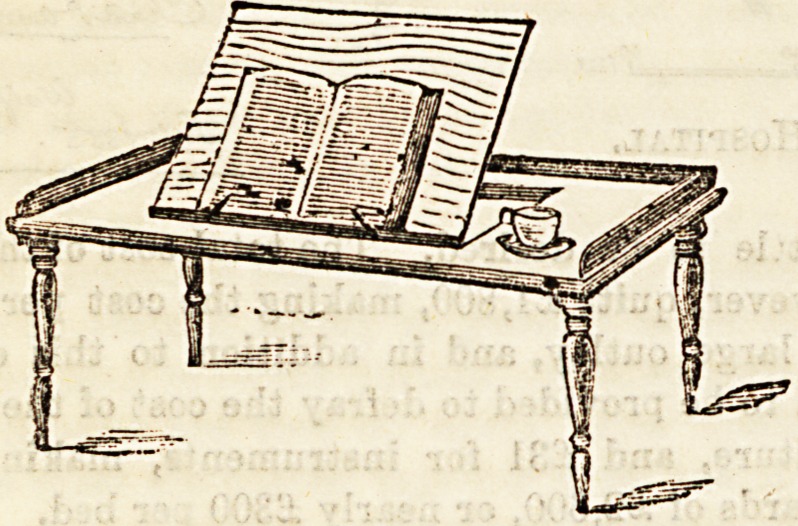


**Figure f2:**